# Methodology Establishment and Application of VITEK Mass Spectrometry to Detect Carbapenemase-Producing *Klebsiella pneumoniae*

**DOI:** 10.3389/fcimb.2022.761328

**Published:** 2022-02-11

**Authors:** Haoyun Lin, Zhen Hu, Jinsong Wu, Yuemei Lu, Jine Chen, Wenyuan Wu

**Affiliations:** Department of Clinical Laboratory, Shenzhen People’s Hospital (The Second Clinical Medical College, Jinan University; The First Affiliated Hospital, Southern University of Science and Technology), Shenzhen, China

**Keywords:** VITEK MS, carbapenemase-producing *Klebsiella pneumoniae*, meropenem, MEM, imipenem, IPM, MALDI-TOF MS, hydrolysis experiment

## Abstract

The ability of VITEK mass spectrometry (MS) in detection of bacterial resistance is currently under exploration and evaluation. In this study, we developed and validated a VITEK MS method to rapidly test carbapenemase-producing *Klebsiella pneumoniae* (CPKP). Solvents, antibiotic concentrations, crystal conditions and times, centrifugation speeds, and other factors were optimized to design a rapid sample pretreatment process for CPKP detection by VITEK MS. The related parameters of the mass spectrum were adjusted on the instrument to establish an CPKP detection mode. 133 clinically isolated strains of CPKP in the microbiology laboratory at the Shenzhen People’s Hospital from 2004 to 2017 were selected for accuracy evaluation. The fresh suspected strains from the microbiology laboratory in 2020 were used to complete the clinical verification. Two antibiotics, meropenem (MEM) and imipenem (IPM), were used as substrates. These two substrates were incubated with suspected CPKP, and the results were obtained by VITEK MS detection. Using this method, different types of CPKP showed different detection results and all the CPKP strains producing KPC-2 and IMP-4 carbapenemase were detected by VITEK MS. Thus, VITEK MS can be used for rapid detection of CPKP, especially for some common types of CPKP. This method provides high accuracy and speed of detection. Combined with its cost advantages, it can be intensely valuable in clinical microbiology laboratories after the standard operating procedures are determined.

## 1 Introduction

Antimicrobial resistance (AMR) is one of the top 10 threats to human health and is an important clinical practical problem that needs to be urgently addressed. The mortality and morbidity of patients caused by AMR also affect global public health and increase the associated economic costs because of its healthcare burden. *Klebsiella pneumoniae* is one of the most common pathogens in clinic. Based on data obtained from the China Antimicrobial Resistance Surveillance System (CARSS) in 2019, *Klebsiella pneumoniae* ranked second only to Escherichia coli among the major clinical isolates (China Antimicrobial Resistance Surveillance system, 2020). During the 14 years from 2005 to 2019, the drug resistance rate of clinically isolated *Klebsiella pneumoniae* to carbapenems increased significantly. The resistance rates of *Klebsiella pneumoniae* to imipenem (MEM) and meropenem (IPM) increased significantly than those of other Enterobacteriaceae bacteria, with an increase from 3.0% to 25.3% and 2.9% to 26.8% from 2005 to 2019, respectively (HU et al., 2020). In addition, the mortality rate from detection of carbapenem-resistant *Klebsiella pneumoniae* (CRKP) infection is 42.14%, which is a serious threat to public health (Xu et al., 2017). There are several drug resistance mechanisms that make *Klebsiella pneumoniae* resistant to carbapenems, but the most common is carbapenemase production (Centonze et al., 2018). Carbapenemases are divided into three major classes with class A, B, and D β-lactamases, according to the Ambler classification system. In the carbapenemases produced by *Klebsiella pneumoniae*, class A mainly include *Klebsiella pneumoniae* carbapenemases (KPC); class B or metallo-β-lactamases mainly include Verona integron–encoded metallo-β-lactamase (VIM), imipenemase (IMP), and New Delhi metallo-β-lactamase (NDM); or class D oxacillinases mainly include oxacillinase-48 (OXA-48) (Nordmann et al., 2011).

At present, the detection of carbapenem resistance in the laboratory is mainly based on phenotype detection methods. The modified carbapenem inactivation method (mCIM) and EDTA-modified carbapenem inactivation method (eCIM) have been introduced in M100 28th. However, the time barrier of extra overnight culture for mCIM and eCIM prevents their use in clinical microbiology laboratories. The Carba NP test recommended by the Clinical and Laboratory Standard Institute (CLSI) can get results within 2 h, but it can only be used for epidemiological or infection prevention purposes (Clinical and Laboratory Standards Institute (CLSI), 2019). This test is not currently recommended for routine use because it lacks good sensitivity for detection of OXA-48-like producers (Lutgring et al., 2018) and KPC-positive strains with low carbapenem MICs cannot be detected in CLSI studies. Genotyping for carbapenem resistance is also beginning to flow into the clinic, but its high cost, long processing time, and heavy informatics burden remain controversial (Liang et al., 2019). For example, PCR tests can only detect known drug resistance genotypes, but detection of new enzyme variants is barely detectable. Xpert Carba-R assay, a commercial rapid PCR-based test, can detect and differentiate five carbapenemase genotypes including VIM, NDM, IMP, KPC, and OXA-48 within 1 h. However, it is expensive for each testing with around €50–55 per test (Bogaerts et al., 2020). Other detection methods, including immunoassay, determination of metabolites, special medium inoculation, and mass spectrometry (MS), are still in the clinical exploratory stage. Common types of carbapenemases can be determined by rapid immunochromatography in immunoassay. For example, Carba 5 assay can detect VIM, NDM, IMP, KPC, and OXA-48 within 15 min, while it costs €28 per assay (Bogaerts et al., 2020). In addition, several studies have indicated that the Carba 5 assay is not sensitive enough for detection of IMP or NDM types (Hopkins et al., 2018; Baeza et al., 2019; Yoon et al., 2021). Thus, a method for detection of carbapenem resistance with relatively low cost and a short detection time window is needed.

MALDI-TOF MS, as a mature soft ionization mass spectrometry technology, has been widely used in the field of microbial identification. MALDI-TOF MS is used not only for the identification of common pathogens but also for the rapid bacterial identification including mycobacteria, filamentous fungi, yeast-like fungi, actinomycetes, and other rare bacteria. The MALDI-TOF MS platforms currently play an important role in the field of microbiological diagnostics.

Two commercial MALDI-TOF MS systems, the VITEK MS and the MALDI Biotyper, are approved by the US Food and Drug Administration (FDA) and are widely used. Previous studies have reported that MALDI-TOF MS can be used for identification of CRKP (Xu et al., 2017; Centonze et al., 2018; Zhang et al., 2019; Huang et al., 2020), MRSA (Kim et al., 2019; Wang H.-Y. et al., 2021), VRE (Griffin et al., 2012; Sabença et al., 2020), and other strains (Sparbier et al., 2012; Kawamoto et al., 2019), but there are only few studies and the conclusions are inconsistent (Lasch et al., 2014; Brackmann et al., 2020; Paskova et al., 2020; Gato et al., 2021).

The aim of this study is to establish a standard operating procedure for rapid detection of CPKP using VITEK MS, so as to complete the preliminary screening of carbapenem resistance in a short time window while obtaining the bacterial identification results. A commercial MALDI-TOF MS-based VITEK MS was used to detect clinically isolated 133 strains of CPKP. Two carbapenem antibiotics, MEM and IPM, were used as substrates through the basic principle of chemical changes of antibiotics under the action of carbapenemases.

## 2 Materials and Methods

### 2.1 Bacterial Strains

#### 2.1.1 Bacterial Strains Used for Method Establishment and Accuracy Evaluation

133 strains of CPKP were isolated from the microbiology laboratory of Shenzhen People’s Hospital from 2004 to 2017. All of these strains obtained antimicrobial resistance data through whole-genome sequencing (WGS). Duplicate strains in the same case were removed.

#### 2.1.2 Bacterial Strains Used for Clinical Validation

The microbiology laboratory of Shenzhen People’s Hospital tested 21 strains suspected to be CRKP from January 2020 to February 2021. 21 *Klebsiella pneumoniae* strains were screened for antimicrobial susceptibility testing (AST) by VITEK 2 (bioMerieux, Marcy l’Etoile, France), and the minimal inhibitory concentrations (MIC) of MEM and IPM showed that mediator or resistance strains were included as clinical verification strains. These strains were mainly from the intensive care unit, neurosurgery unit, respiratory intensive care unit, and other departments. Duplicate strains in the same case were removed.

### 2.2 Carbapenemase Detection

#### 2.2.1 Preliminary Screening Method for AST

##### 2.2.1.1 Disc Diffusion Testing

Carbapenem non-susceptibility was suspected when the zone diameter on disc diffusion testing was ≤22 mm for imipenem or meropenem disks.

##### 2.2.1.2 MIC by VITEK 2

Carbapenem non-susceptibility was also suspected when MICs of MEM or IPM ≥ 2 μg/ml was detected by VITEK 2. MICs of carbapenems were determined by the VITEK 2 test using the AST 335 card (bioMerieux, Marcy l’Etoile, France), which includes IPM (range, 1 to 16 μg/ml) and MEM (range, 0.25 to 16 μg/ml), according to the manufacturers’ recommendations.

#### 2.2.2 Broth Micro-Dilution Method

MIC of 133 CPKP strains and 21 clinically validated strains were detected by broth microdilution method (BMD) and performed according to CLSI guidelines. 0.5 McFarland suspension of the isolates was prepared and diluted 100-fold with cation-adjusted Mueller–Hinton broth (CAMHB). 50 μl of the bacterial suspension was seeded into a 12-well plate containing 50 μl of CAMHB with serial concentrations of antibiotics. The final inoculum of the bacteria was approximately 5 × 10^5^ CFU/ml. The final concentrations of IPM and MEM were 32, 16, 8, 4, 2, 1, 0.5, 0.25, 0.125, 0.064, 0.032, and 0.0016 μg/ml. The suspension was cultured at 35°C for 18–20 h.

#### 2.2.3 mCIM/eCIM Testing

Suspected carbapenemase production was detected in 133 CPKP strains and 21 clinically validated strains by mCIM and eCIM.

##### 2.2.3.1 mCIM

For each isolate to be tested, 1 µl loopful of bacteria was emulsified from an overnight blood agar plate in 2 ml tryptic soy broth (TSB) and vortexed. A 10-µg MEM disk was added to each tube. The entire disk was immersed in the suspension and incubated at 35°C in ambient air for 4 h. 0.5 McFarland suspension of *E. coli* ATCC 25922 was prepared in saline and inoculated with a Mueller–Hinton agar (MHA) plate within 15 min. The plates were dried, and then MEM disks were added. The MEM disk was removed, and excess liquid from the disk was expelled and then placed on the Mueller–Hinton agar (MHA) plate. The MHA plates were inverted and incubated at 35°C in ambient air for 18–24 h. The zones of inhibition were measured for the routine disk diffusion method.

##### 2.2.3.2 eCIM

20 µl of 0.5 M EDTA was added to the 2-ml TSB tube to obtain a final concentration of 5 mM EDTA. The same procedure as for mCIM was followed. The mCIM and eCIM tubes were processed in parallel. The MEM disks from the mCIM and eCIM tubes were placed on the same MHA plate inoculated with the MEM-susceptible *E. coli* ATCC 25922 indicator strain. The interpretations were performed according to the experimental procedures in CLSI M100-S29 (Clinical and Laboratory Standards Institute (CLSI), 2019).

#### 2.2.4 Whole-Genome Sequencing, Assembly, and Data Analysis

Whole-genome sequencing was performed on 133 CPKP strains that completed the preliminary screening and phenotypic detection of carbapenemase. The whole-genome DNA of bacteria was extracted by Tiangen Bacterial Genome DNA Extraction Kit (Tiangen, Beijing, China). Sequencing libraries were prepared using NextEra Ultra. Two-terminal sequencing was performed on an Illumina II High-throughput Sequencing Platform and PE150 Sequencing Strategy. The whole-genome sequencing was commissioned by Shenzhen Haiyi Times Genomics. The genome of sequencing data was assembled by SOAPdenovo, and then the inner hole of the assembly result was repaired by GapCloser software. GeneMarks software was used to predict coding genes. The carbapenemase resistance genes were searched through the CARD database and ResFinder in CGE, and the annotations of the functional database were compared by BLAST software. Carbapenem resistance genes were screened out according to the annotation results of WGS.

#### 2.2.5 Immunochromatographic Test

NG-Test Carba 5 immunochromatographic assay (NG Biotech, Guipry, France) was used as a combination test for clinical validation. According to the selection test strip instructions, KPC-, NDM-, IMP-, VIM-, and OXA-48-type carbapenemases were detected in the range. Briefly, 150 μl extraction buffer was dispensed in the microtube. 3 pure colonies with a loop were touched and suspended in the microtube containing 150 µl of extraction buffer and vortexed to homogenize. 100 μl of the prepared mixture was added in the sample well. The result was read at 15 min according to the manufacturers’ recommendations (Han et al., 2021).

#### 2.2.6 Rapid Detection of CPKP Based on VITEK MS

##### 2.2.6.1 Experimental Subject

###### 2.2.6.1.1 Positive Strain

133 clinically isolated CPKP strains were used as positive strains to participate in the establishment and detection based on VITEK MS. All positive strains were blaKPC-2 (77 strains), blaIMP-4 (43 strains), blaIMP-26 (3 strains), blaNDM-1 (9 strains), and blaIMP-4+blaKPC-2 (1 strains). 21 clinically isolated strains suspected to be CRKP were also used as positive strains for clinical validation.

###### 2.2.6.1.2 Negative Control Strain

ATCC13883 (carbapenem sensitive *Klebsiella pneumoniae*) was used as a negative control strain. The quality control strain of *Escherichia coli* ATCC8739 was used for calibration of the VITEK MS system.

###### 2.2.6.1.3 Positive Control Strain

ATCCBA-1705 (KPC positive *Klebsiella pneumoniae*) was used as a positive control strain.

###### 2.2.6.1.4 Drug Quality Control

2.0 mmol/l MEM solution was prepared with 20 mM Tris–HCl pH 6.8 solution; 1.0 mg/ml IPM solution was prepared with 0.45% NaCl.

###### 2.2.6.1.5 Reagent Blank Quality Control

20 mM Tris–HCl pH 6.8 solution and 0.45% NaCl solution were prepared.

##### 2.2.6.2 Sample Pretreatment

###### 2.2.6.2.1 MEM Hydrolysis Experiment

The strains were incubated overnight on 5% Columbian sheep blood plates at 37°C. Then, pure colonies and 1 ml MEM solution (2 mmol/l) were selected to prepare 3.5 McFarland bacterial suspension. The bacterial suspension was centrifuged at 13,000 rpm for 3 min. The supernatant was discarded, and 40 μl MEM solution (2 mmol/l) and 10 μl 0.45% NaCl solution were added into the precipitate and mixed thoroughly and then incubated at 37°C for 2 h. After the incubation, the tubes were centrifuged for 3 min at 13,000 rpm. 1 μl of the supernatant was applied to each target spot and left to dry with cold air at 19°C for 15 min, then covered with 1 μl of CHCA matrix solution to dry and form crystals and performed using VITEK MS (RUO mode).

###### 2.2.6.2.2 IPM Hydrolysis Experiment

The strains were incubated overnight on 5% Columbian sheep blood plates at 37°C. Pure colonies were mixed with 1 ml IPM solution (1.0 mg/ml) to prepare a bacterial suspension with turbidity of 3.5 McFarland. The bacterial suspension was centrifuged at 13,000 rpm for 3 min, and the supernatant was discarded. 50 μl IPM solution (1.0 mg/ml) was added into the precipitate and then incubated at 37°C for 20 min. After the incubation, the tubes were centrifuged for 3 min at 13,000 rpm. 1 μl of the supernatant was applied to each target spot and then covered with 1 μl of CHCA matrix solution to dry and form crystals and performed using VITEK MS (RUO mode).

##### 2.2.6.3 Mass Spectrometry Parameter Setting

MALDI-TOF MS was performed with VITEK MS using a 48-spot target plate. The linear detector (LD) value was set to 2,700, the laser power was set to 68, and the mass range was set to 200–600. In graphic analysis, it was set to 370–430 for the MEM hydrolysis experiment, 280–500 for the IPM hydrolysis experiment. The smoothing method is set to off, the display contents is set to stack, and the options is set to Angle 90.

##### 2.2.6.4 Result Judgment

A result was interpreted as positive for carbapenemase production if the peaks for MEM (384 m/z) or IPM (300 m/z and 489 m/z) disappeared completely during the incubation time. If the peak of MEM (384 m/z) or IPM (300 and 489 m/z) still exists after testing, it was judged as a negative result, under the condition of normal quality control.

In our MEM hydrolysis assay, obvious peak values of MEM degradation (358 Da, the decarboxylated product) as well as its sodium salts (380 Da) were observed, as shown in **Figure 2A**. However, MEM was prone to degradation during the transport and configuration of reagents (Calderaro et al., 2017), due to the special molecular structure (more chiral centers lead to structural instability). VITEK MS is unable to calculate the corresponding ratio (logRQvalue), as well as the intensities of hydrolyzed and intact MEM. We therefore selected the disappearance of the original peak value of MEM as the judgment parameter of CPKP detection.

## 3 Results

### 3.1 The Experimental Principle of Detecting CPKP Based on VITEK MS

We screened whether *Klebsiella pneumoniae* produced carbapenemases by detecting the molecular weight changes of carbapenems using VITEK MS. Considering the instability of MEM and IPM, the structures of these two compounds were changed by the pretreatment process and the subsequent laser shock effect. Importantly, their original molecular weights, namely, the original peak value, could be detected under appropriate experimental conditions without the effect of carbapenemase. However, when carbapenemase was present, both compounds degraded completely due to enzymatic interactions. **Table 1** lists the compositions and molecular weights of related compounds for MEM and IPM. During the ionization process, antibiotic molecules usually add a proton, which increases the molecular mass by 1. MEM (molecular mass about 383 Da) and IPM (molecular mass about 299 Da) changed into 384 and 300 m/z in the mass spectrum after the ionization process. We also found that during the ionization process of MEM, one or two sodium ions may be added, which is the same as the study of Sparbier et al. (Sparbier et al., 2012). In addition to adding a proton, IPM also forms a mixture with the matrix liquid (CHCA) during the ionization process, and the molecular weight of the mixture is 489.4, which is consistent with the results of Oviaño et al. (Oviaño et al., 2017).

**Table 1:** Structural determination of MEM and IPM by MALDI-TOF MS^a^.

Antibiotic	Compounds and its *molecular weight*s				
	MW*^b^* (g/mol)	[M+H]^+^	[M+Na]^+^	[M+2Na]^+^	[M+C*^c^* +H]^+^
MEM	383.4	384.5	406.5	428.5	
IPM	299.4	300.4			489.4

^a^See references (Sparbier et al., 2012; Oviaño et al., 2017).

^b^MW, molecular weight.

^c^Imipenem is complexed with the matrix (α-cyano-4-hydroxy-cinnamic acid), resulting in a peak that is the summation of their molecular masses.

### 3.2 Establishment and Optimization of the VITEK MS Method for Rapid Detection of CPKP

The experimental steps using VITEK MS to detect CPKP are divided into before and after entering the mass spectrometer. We optimized these steps to safeguard the standardized operating procedures.

#### 3.2.1 Pretreatment Process of Specimen Before Testing on VITEK MS

**Figure 1:**
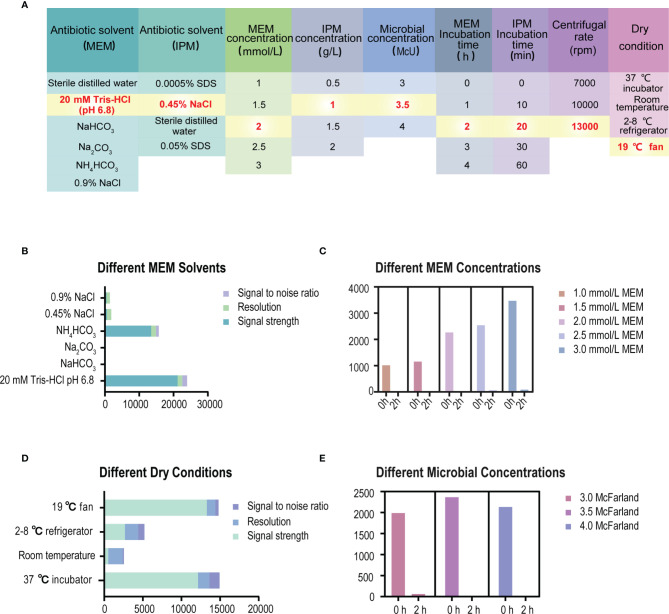
Experimental conditions for specimen pretreatment of detecting CPKP based on VITEK MS. **(A)** List of specific experimental parameters. **(B)** The parameters of the MEM mass spectrum under different solvent conditions. **(C)** The parameters of the MEM mass spectrum under different MEM concentrations. **(D)** The parameters of the MEM mass spectrum under different dry conditions of co-crystalline film formation. **(E)** The parameters of the MEM mass spectrum under different microbial concentration conditions.

**Figure 2:**
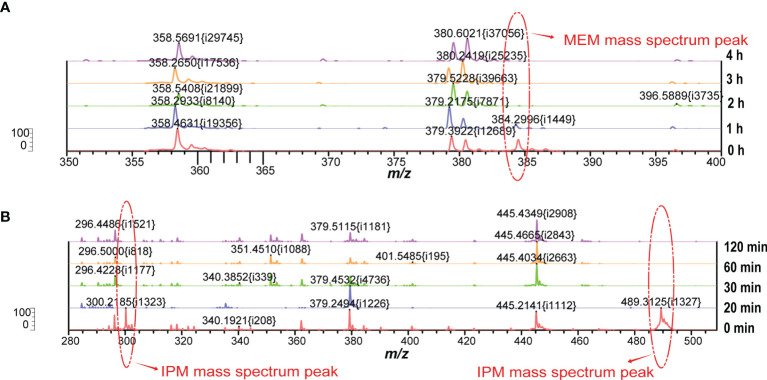
Changes of peak mass spectra of MEM or IPM at different incubation times. **(A)** The peak value of the MEM mass spectrum at different incubation times. **(B)** The peak value of IPM mass spectrum at different incubation times.

**Figure 3:**
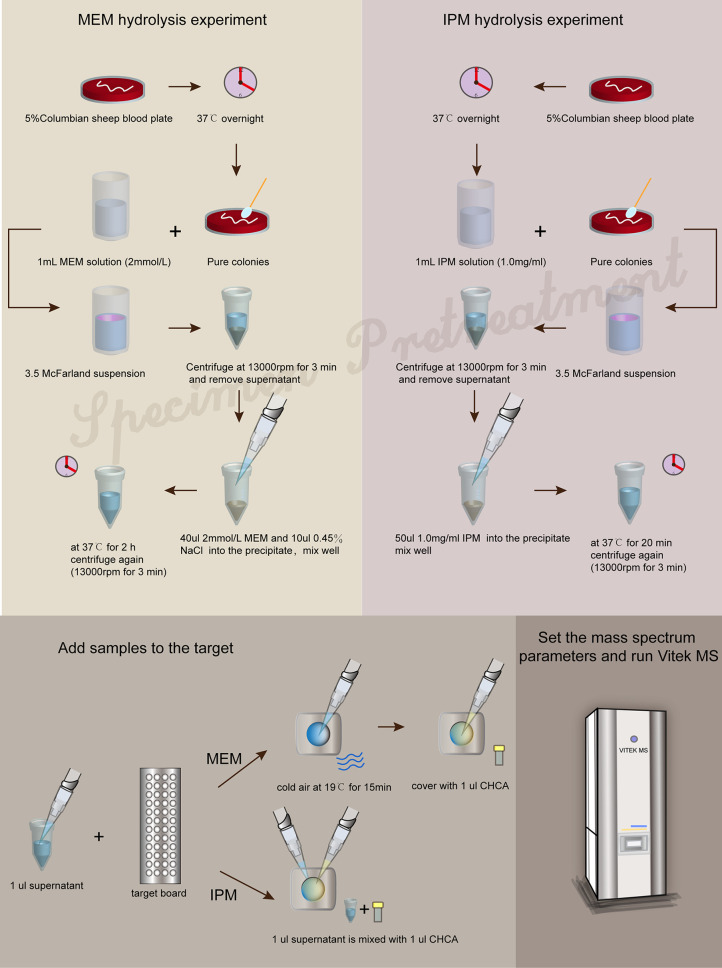
Flowchart of CPKP detection by VITEK MS.

The formation of a co-crystalline film containing sample and matrix solution is the key problem that needs to be solved in the pretreatment. The factors influencing the formation of co-crystalline films were evaluated (**Figure 1A**). Dissolving MEM or IPM in an appropriate solvent to ensure its structural stability was the first consideration. It was found that MEM maintained the best stability in the solution of 20 Mm Tris–HCl at pH 6.8, where the signal strength reaches the maximum value (**Figure 1B**). However, IPM did not need additional chemical reagents as the solvent, and it met the detection requirements when 0.45% NaCl was selected as the solvent. The optimal signal intensity was obtained when the turbidity of the bacterial liquid was 3.5 McFarland standard (**Figure 1E**) and the centrifugation speed was 13,000 rpm. At the same time, cold air drying at 19°C for 15 min was used to form the co-crystalline film of bacteria and matrix, ensuring the structural stability of MEM to the greatest extent (**Figure 2D**). In order to get the peak value of the mass spectrum with high signal intensity, the formation order of the co-crystalline films was also different when MEM or IPM was used as a substrate. The optimal concentrations of these two antibiotics were different, where MEM was 2 mmol/l (**Figure 1C**) and IPM was 1.0 mg/ml. In addition, there were significant differences in the incubation time between the two antibiotics. Carbapenemases produced by CPKP make the original peak value of MEM (384 m/z) disappear after MEM was incubated for 2 h with bacterial solution. On the other hand, IPM and CPKP were incubated for only 20 min and the original peak value of IPM (300 m/z and 489 m/z) disappeared (**Figures 2A, B**). The related steps have been embodied in MEM and IPM hydrolysis experiments. By optimizing the parameters of the pretreatment experiment, a flowchart of CPKP detection using VITEK MS was established in this experiment, so as to more clearly show the differences in the pretreatment steps when two different substrates (MEM and IPM) were used. **Figure 3** shows the detailed process of detecting CPKP by VITEK MS using MEM or IPM as a substrate.

#### 3.2.2 The Setting of Mass Spectrum Parameters Before Detection

All experiments were performed in the RUO mode of VITEK MS, allowing for the adjustment of mass spectral parameters. Only an appropriate laser power can ensure MEM or IPM on the target to fully act and reach the detector through the linear flight tube. After optimization, appropriate parameters for MS detection were set up, as shown in **Table 2**.

**Table 2:** Mass spectrum parameter settings for CPKP detection.

Detection mode	Testing				Graphic analysis			
	Antibiotic	Linear detector	Power	Mass range (detection)	Mass range (analysis)	Smoothing method	Display contents	Options
RUO	MEM	2700	68	200–600	370–430	off	stack	Angle 90
	IPM	2700	68	200–600	280–500	off	stack	Angle 90

### 3.3 Accuracy Evaluation of Rapid Detection of CPKP on VITEK MS

#### 3.3.1 Information About the Strain Used for Detection

The CPKP strains preserved from 2004 to 2017 were recovered and sequenced by WGS to obtain the relevant drug resistance gene information. In addition, the phenotype of carbapenemase was also tested according to mCIM/eCIM testing recommended by CLSI. These 133 CPKP samples were derived from sputum (50), blood (24), urine (21), catheter (5), drainage fluid (9), bronchoalveolar lavage fluid (5), tracheal aspirate (5), cerebrospinal fluid (2), ascites (2), bile (1), pleural fluid (1), pus (1), secretions (2), throat swabs (3), puncture fluid (1), and tissues (1). The 133 strains were sorted according to MIC of MEM and IPM, year, department, and antimicrobial-resistant gene. The details are shown in **Table 3**. The carbapenemase phenotypic results of mCIM and eCIM of 133 CPKP strains were consistent with the classification of carbapenemase genotype results annotated by WGS (**Supplementary Material**).

**Table 3:** MIC values of 133 CPKP strains and their carbapenem resistance genes.

Department	Year	Specimen source	Drug-resistant gene	VITEK 2-MIC (MEM)	VITEK 2- MIC (IPM)	BMD-MIC (MEM)	BMD-MIC (IPM)
CCU	2017	Sputum (2);	KPC-2	≥16	≥16	>32	32(1); >32(1)
ECU	2017	Tissue (1); abdominal fluid (1); drain (1)	KPC-2	≥16	≥16	>32	32(1); >32(2)
Ent	2016	Cerebrospinal fluid (1)	KPC-2	≥16	≥16	>32	32
Gastrointestinal surgery	2016	Drain (1); catheter (2)	KPC-2	≥16	≥16	>32	16(1); 32(2)
Geriatrics	2009; 2017	Sputum (1); urine (2)	IMP-4 (1); KPC-2 (2)	≥16	≥16	8(2); >32(1)	4; 8; 16
Gynaecology	2015	Catheter (1)	NDM-1	≥16	≥16	>32	>32
Hematology	2017	Blood (1)	KPC-2	≥16	≥16	>32	>32
Hepatobiliary surgery	2010; 2016	Pus (1); blood (1)	IMP-4+KPC-2; NDM-1	8;≥16	≥16	16; >32	8; >32
ICU	2008; 2010–2012; 2014–2017	Bile (1); blood (6); broncholveolr lvge fluid (4); catheter (1); drain (6); pleural effusion (1); sputum (15); urine (3)	IMP-26 (2); IMP-4 (5); KPC-2 (29); NDM-1 (1)	4 (1); ≥16 (36)	8 (1); ≥16 (36)	8 (1); 16 (8); 32 (4);>32 (24)	4 (3); 8 (8); 32 (18); >32 (8)
Neonate department	2008; 2010–2014; 2017	Blood (8); tracheal aspirate (5); catheter (1); sputum (1); throat swab (2); urine (2) bronchoalveolar lavage (1); puncture fluid (1)	IMP-4 (21);	1 (1); 4 (6); ≥16 (14)	0.5 (1); 8 (8); ≥16 (12)	2 (1); 4 (2); 8 (16); 16 (2)	0.5 (1); 2 (5); 4 (13); 8 (2)
Nephrology	2009; 2017	Sputum (1); urine (1)	IMP-4 (1); KPC-2 (1);	≥16	≥16	8 (1); >32 (1)	4 (1); 32 (1)
Neurology	2016; 2017	urine (4)	NDM-1 (1); KPC-2 (4);	≥16	≥16	16 (1); 32 (1);>32 (2)	8 (1); 32 (3)
Neurosurgery	2004; 2008–2011; 2013; 2016; 2017	Abdominal fluid (1); cerebrospinal fluid (1); drain (1); secretion (1); sputum (10); throat swab (1); urine (7)	IMP-4 (9);IMP-26 (1); KPC-2 (12);	≥16	8 (4); ≥16 (18)	4 (2); 8 (5); 16 (6);>32 (9)	4 (8); 8 (5); 32 (6);>32 (3)
Obstetric	2016	Secretion (1)	NDM-1	≥16	≥16	>32	32
Respiratory	2016; 2017	Sputum (5); blood (1)	KPC-2	≥16	≥16	>32	32 (2); >32 (4)
Rheumatology	2017	Throat swab (1); urine (1)	IMP-4; KPC-2	≥16	≥16	>32	32
RICU	2010; 2016; 2017	Sputum (13); blood (2); urine (1)	IMP-4 (5); KPC-2 (11)	1 (3); 4 (1); ≥16 (12)	≤0.25 (1); 8 (2); ≥16 (13)	2 (1); 8 (3); 16 (1); 32 (1); >32 (10)	1 (1); 2 (3); 4 (1); 16 (2); 32 (5); >32 (4)
The Emergency Ward	2013; 2014; 2016; 2017	Sputum (1); blood (5);	KPC-2 (2); NDM-1 (4)	≥16	≥16	32 (3); >32 (3)	8 (1); 16 (2); 32 (1);>32 (2)

The numbers in brackets indicate the number of CPKP strains. The MIC value is expressed in μg/mL.

#### 3.3.2 Accuracy Evaluation of Detection Results Using MEM as a Substrate

**Figure 4:**
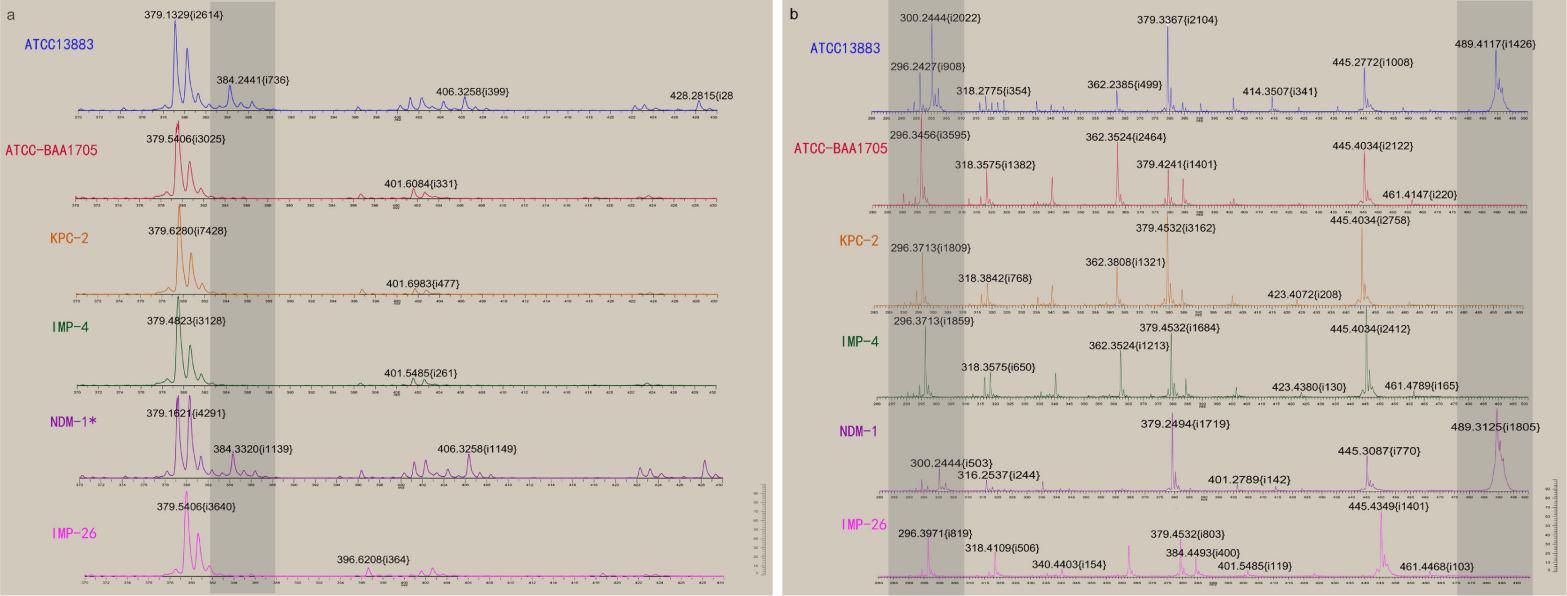
Detection results of CPKP were obtained by VITEK MS**. (A)** The changes of the mass spectrum of carbapenemase were detected when MEM was used as a substrate for hydrolysis. NDM-1* represents that NDM-1 genotype CPKP was not detected by VITEK MS. **(B)** The changes of the mass spectrum of carbapenemase were detected when IPM was used as a substrate for hydrolysis.

133 strains of CPKP were pretreated by MEM hydrolysis experiment and tested by VITEK MS (**Figure 4A** and **Table 4**). After a 2-h incubation, 94.7% (126/133) of CPKP showed that the MEM (384 m/z) peak disappeared compared with the enzyme-negative strains. However, the results showed some differences between various antimicrobial-resistant genotypes. This may be related to the amount and types of carbapenemase released by different antimicrobial-resistant genotypes. After 2 h of incubation, the MEM (384 m/z) peak disappeared in all CPKP strains with the KPC-2 genotype (77/77) and IMP-4 genotype (43/43). However, the results of NDM-1 strain were found to be unstable, and the original peak value of MEM (384 m/z) disappeared only in 2 NDM-1 strains (2/9). When CPKP with more than two carbapenemase-resistant genotypes were detected by WGS, good results could be obtained in the MEM hydrolysis experiment by VITEK MS.

**Table 4:** Accuracy evaluation of detection results using MEM or IPM as a substrate.

Detection results using MEM as substrate				Detection results using IPM as substrate			
Type	WGS	VITEK MS	Accuracy	Type	WGS	VITEK MS	Accuracy
KPC-2	77	77		KPC-2	77	77	
IMP-4	43	43		IMP-4	43	43	
IMP-26	3	3		IMP-26	3	3	
NDM-1	9	2		NDM-1	9	0	
KPC-2+IMP-4	1	1		KPC-2+IMP-4	1	1	
Total	133	126	94.7%	Total	133	124	93.2%

#### 3.3.3 Accuracy Evaluation of Detection Results Using IPM as a Substrate

133 strains of CPKP were pretreated by IPM hydrolysis experiment and tested by VITEK MS (**Figure 4B** and **Table 4**). After 20 min of incubation, 93.2% (124/133) of CPKP showed that the IPM (300 and 489 m/z) peak disappeared compared with the enzyme-negative strains. Among them, the peak value of IPM (300 and 489 m/z) disappeared after the IPM hydrolysis experiment when the detected strain was KPC-2 (77/77) or IMP-4 (43/43). When two carbapenemase-resistant genotypes in the strain were detected by WGS, the peak (300 and 489 m/z) disappearance could be detected on VITEK MS. These results were consistent with the results obtained from the MEM hydrolysis experiment. For strains of NDM-1 (9/133) genotypes, the results of the IPM hydrolysis experiment were also unstable.

### 3.4 Clinical Validation Results

The 21 strains of *Klebsiella pneumoniae* isolated in the microbiology laboratory of Shenzhen People’s Hospital in 2020 were used as clinical verification strains. Carbapenemase was detected in 21 clinically validated strains by phenotype detection, gold-standard immunoassay, VITEK MS, and PCR, respectively. The detailed results of clinical validation are shown in **Table 5**. Among the 21 clinically validated strains, the VITEK MS detection results of 20 CPKP strains were consistent with MEM or IMP as a substrate, except No. 54538, where the detection results of MEM as a substrate were not the same with those of VITEK MS using IPM as a substrate. The genotype of carbapenemase production was therefore determined to be VIM-1 by WGS. VITEK MS results of the other 20 clinically validated strains were consistent with PCR and immunogold results. In the clinical validation results, the sensitivity and specificity of the MEM hydrolysis test for carbapenemase detection by VITEK MS were 100% and 100% and the sensitivity and specificity of the IPM hydrolysis test were 95.2% and 100%, if the PCR method is used as a reference method.

**Table 5:** Carbapenemase detection results of 21 clinically validated strains.

Strain no.	Disk-MEM	Disk-IPM	BMD-MEM	BMD-IPM	VITEK 2-MEM	VITEK 2-IPM	mCIM	eCIM	Carbapenemase	Metallo-β-lactamase	Immunogold	PCR	WGS	VITKE MS	VITKE MS
	(mm)	(mm)	(μg/ml)	(μg/ml)	(μg/ml)	(μg/ml)								(MEM)	(IPM)
52653	10/R	13/R	32/R	32/R	≥16/R	≥16/R	6	6	+	–	KPC	KPC	KPC-2	+	+
52848	6/R	6/R	16/R	16/R	≥16/R	≥16/R	6	19	+	+	NDM	NDM	NDM-1	+	+
53123	6/R	8/R	>32/R	32/R	≥16/R	8/R	6	20	+	+	NDM	NDM	NDM-5	+	+
53262	6/R	7/R	>32/R	32/R	≥16/R	≥16/R	6	6	+	–	KPC	KPC	KPC-2	+	+
54080	6/R	7/R	32/R	16/R	≥16/R	≥16/R	6	20	+	+	IMP	IMP	IMP-4	+	+
54538	6/R	13/R	1/S	2/I	1/S	≥16/R	6	19	+	+	VIM	VIM	VIM-1	+	–
54645	11/R	13/R	>32/R	32/R	≥16/R	≥16/R	6	6	+	–	KPC	KPC	KPC-2	+	+
55120	6/R	13/R	32/R	32/R	≥16/R	≥16/R	6	20	+	+	NDM	NDM	NDM-1	+	+
55233	6/R	6/R	>32/R	32/R	≥16/R	≥16/R	6	6	+	–	KPC	KPC	KPC-2	+	+
55301	14/R	17/R	8/R	4/R	≥16/R	2/I	6	20	+	+	IMP	IMP	IMP-4	+	+
55841	6/R	6/R	>32/R	32/R	≥16/R	≥16	6	6	+	–	KPC	KPC	KPC-2	+	+
55867	6/R	6/R	>32/R	32/R	≥16/R	≥16	6	6	+	–	KPC	KPC	KPC-2	+	+
56949	6/R	6/R	>32/R	>32/R	≥16/R	≥16	6	19	+	+	IMP	IMP	IMP-4	+	+
57025	6/R	6/R	32/R	32/R	≥16/R	≥16	6	21	+	+	NDM	NDM	NDM-1	+	+
57139	6/R	6/R	>32/R	32/R	≥16/R	≥16	6	19	+	+	NDM	NDM	NDM-1	+	+
57637	6/R	6/R	>32/R	32/R	≥16/R	≥16	6	7	+	–	KPC	KPC	KPC-2	+	+
52664	19/R	32/S	0.016/S	0.125/S	≤0.25/S	≤0.25/S	23	22	–	–	–	–	–	–	–
52474	21/I	31/S	2/I	1/S	1/S	≤0.25/R^#^	21	21	–	–	–	–	–	–	–
56004	22/I	28/S	0.5/S	0.25/S	1/S	≤0.25/R^#^	21	21	–	–	–	–	–	–	–
52986	23/S	32/S	0.064/S	0.5/S	≤0.25/S	2/I	20	21	–	–	–	–	–	–	–
52191	22/I	25/S	0.5/S	1/S	1/S	1/R^#^	20	21	–	–	–	–	–	–	–

The values before “/” are the diameters of inhibitory zone or the MIC values, and the values after “/” are sensitive (S), intermediate (I), and resistance (R). “+” means positive result; “-” means negative result. R^#^ stands for drug resistance determined by VITEK 2 Advanced Expert System (AES)*.

## 4 Discussion

In this study, we performed a rapid screening of CPKP using MALDI-TOF MS-based VITEK MS, which demonstrated the ability of MALDI-TOF MS to screen for AMR. Our results show that CPKP can be rapidly and specifically identified by VITEK MS within 20 min to 2 h using IPM or MEM as a substrate. VITEK MS can also be used as an experimental method for routine screening of CPKP. According to the operating procedure in our study, this method can be easily achieved in an ordinary microbiology laboratory. The mass spectrometric matrix used is officially selected CHCA, which was provided by the manufacturer. This study provides an idea for the detection of enzyme-producing AMR, and screening of resistant strains could be achieved by detecting the degradation of antibiotic substrates under the action of antimicrobial resistance enzymes.

MALDI-TOF MS-based methods on detection of bacterial resistance are mainly divided into three categories: (1) detection of characteristic spectrum peaks of antimicrobial-resistant bacteria (Cordovana et al., 2018; Dortet et al., 2018; Li et al., 2019; Dortet et al., 2020); (2) detection of bacterial growth in the presence of antibiotics (Correa-Martínez et al., 2019; Axelsson et al., 2020; Wang G. et al., 2021); and (3) detection of the antimicrobial agent change under the action of enzyme produced from antimicrobial resistant bacteria (Hoyos-Mallecot et al., 2014; Oviaño et al., 2014; Xie et al., 2019). The characteristic peak spectrum associated with AMR is the most ideal indicator to be detected by MALDI-TOF MS in principle. However, some of the results that were thought to be characteristic peaks have been reversed in subsequent studies. This may indicate that the peaks created based on specific groups only apply to the local epidemic strains and are needed to be validated before application in other areas (Wang et al., 2018). A retrospective study used MALDI-TOF MS to observe that the 11,109 m/z peak is corresponding to a specific protein encoded by the pKPQIL plasmid (P019) and can also be used to screen for KPC-producing Enterobacteriaceae (Cordovana et al., 2018). However, a new study has shown that the p019 gene is not exclusively linked to the pKpQil plasmid and it presents in the following plasmids: IncFIB(K)/IncFII(K)/ColRNAI, IncFIB(pQil), IncFIB(pQil)/ColRNAI, IncFIB(pQil)/IncFII(K), IncFIB(K)/IncFII(K), and IncX3. This should be taken into account when using 11,109 m/z as the characteristic peak for KPC screening on MALDI-TOF MS-based method (Gato et al., 2021).

According to the second strategy, microbiologists try to identify antimicrobial-resistant bacteria by exploring how they grow in an antibiotic environment. Some studies distinguish the sensitive bacteria from specific antibiotic resistance by the differences in their growth process through the culture of pathogenic bacteria on isotope-labeled medium (Sparbier et al., 2013; Grey et al., 2021). However, the special medium limitations, background peak interference, and other factors have influenced the development of this method in clinical microbiology laboratories. The detection of bacterial growth in the environment with different antibiotic concentrations to obtain MIC is also an exploration direction through MALDI-TOF MS-based methods, but the MIC for antimicrobial-resistant bacteria may be lower than that for AST by the broth micro-dilution method (Wang G. et al., 2021).

In this study, the detection principle adopted belongs to the third detection strategy as mentioned above. The huge potential of MALDI-TOF MS in the rapid detection of drug resistance has been demonstrated by the enzyme-mediated antibiotic hydrolysis assays (Wang G. et al., 2021). The premise of this method is that the mechanism of antimicrobial resistance in pathogens is inactivated by enzyme lysis/modification. In 2011, enzyme hydrolysis assay was used for the first time to detect CPKP using MALDI Biotyper (Burckhardt and Zimmermann, 2011; Hrabák et al., 2011). Since then, this method has been used in many studies and some of them were tested with the Bruker-introduced MBT STAR-Carba IVD kit and the MBT STAR-Bl IVD software (Hrabák et al., 2012; Sauget et al., 2014; Lasserre et al., 2015; Calderaro et al., 2017; Oho et al., 2021). However, studies on the enzymatic hydrolysis test for CPKP by VITEK MS are lacking (Knox et al., 2014; Cecilia Godoy Carvalhaes et al., 2015; Horseman et al., 2020), and there is no corresponding diagnostic kit and calculation software developed by bioMerieux (Neonakis and Spandidos, 2019; Oviaño and Bou, 2019). In the previous research on the detection of bacterial resistance based on MALDI-TOF MS technology, VITEK MS has been limited to the exploration of microbiologists due to its commercially specified matrix solution and less selection of mass spectrometric adjustment parameters. In our study, we found that VITEK MS could obtain reliable detection results with the matrix solution CHCA and adjustable mass spectrometry parameters. The official matrix solution of VITEK MS is CHCA is believed to be indistinguishable for MEM (384 m/z) and MEM degradable substance (380 m/z) due to its characteristic peak spectrum (379 m/z) (Hrabák et al., 2011; Lasserre et al., 2015). However, our results show that VITEK MS with CHCA has the ability to distinguish between these two peaks.

It is worth mentioning that there is no standardized experimental procedure and protocol for the current detection of CPKP based on MALDI-TOF MS, which is exactly what this study intends to accomplish through VITEK MS, a commercialized MALDI-TOF MS-based method that can be widely used in clinical microbiology laboratories. In addition to the selection of antibiotic substrates, the lack of established and standardized routines for MALDI-TOF MS-based technology in detecting antimicrobial resistance is one of the future challenges of MALDI-TOF MS (Monteferrante et al., 2016; Oviaño and Bou, 2019). The length of incubation time is also one of the key factors determining the development prospects of the MALDI-TOF MS method for detecting CPKP. In previous studies on VITEK MS, imipenem incubation took 4 h as a substrate (Knox et al., 2014). In this study, the incubation time of imipenem could be reduced to 20 min and meropenem can be used as a substrate by incubation for 2 h if the experiment is carried out according to the established standard operating procedures.

VITEK MS can be used to identify the strain species by directly screening CPKP if the identification result is *Klebsiella pneumoniae*, which is faster and more labor cost-effective than the conventional CPKP screening or confirmation in clinical practice. VITEK MS tests for CPKP cost less than €1 per test. The cost of VITEK MS was significantly lower than that of Xpert Carba-R and immunochromatography, two new carbapenemase detection methods that have been used in clinic. In this study, two hydrolysis experiments had some differences in detecting the NDM-1 genotype CPKP based on VITEK MS. None of NDM-1 (9 strains) was detected by hydrolysis experiments based on VITEK MS with IPM as a substrate, while two strains were detected with MEM as a substrate (2/9). However, among the 21 strains clinically verified by us, NDM-1 (4/4) and NDM-5 (1/1) genotype CPKP could be detected by VITEK MS using MEM and IPM substrates. Compared with IPM as a substrate, the hydrolysis experiment with MEM as a substrate has better NDM-1 detection sensitivity in our setting, but due to the small number of these types of experimental strains, further expansion of the experiment is needed. There are also some limitations as there are many drug resistance mechanisms in pathogens. The production of carbapenemase is one of the most common mechanisms of carbapenem resistance in *Klebsiella pneumoniae*. Therefore, this strategy is suitable for the detection of CPKP based on MALDI-TOF MS.

The linear detector in VITEK MS is mainly used to detect proteins and large peptides. The official detection range of the IVD mode is set at 2,000–20,000 Da. It was found that the linear detector also showed a high resolution even for small molecules (0–800 Da). In the process of detecting CPKP based on VITEK MS, the pretreatment process of the specimen is very critical, and the quality of this step determines the final test result. Differences in pretreatment processes and changes in mass spectrum parameters may be the reasons for inconsistent detection results in past studies, excluding the regional influence of strain origin. We therefore standardized and quantified various experimental factors to ensure the smoothness of the experimental process and the stability of the experimental results. In the experiment, we found that the setting of the mass spectrum parameters and the pretreatment process of the strain needed to be accurately controlled in accordance with the experimental links. The operating state and calibration level of VITEK MS are both key factors affecting the results. It is recommended that the calibration of the mass spectrometer will be completed by the instrument engineer before the experiment. Herein, we believe that as long as the following four conditions are satisfied: (1) antimicrobial resistance enzyme exists; (2) appropriate antibiotic substrate; (3) standardized pretreatment steps; and (4) mass spectrometry parameters that meet the standard, we can explore the establishment of a standard operating procedure based on MALDI-TOF MS technology for the detection of this type of AMR.

In this work, hundreds of CPKP strains were successfully detected by VITEK MS. The influences of different solvents, antibiotic concentration, crystal formation conditions and time, centrifugal speed, and other factors on the detection effect were compared in detail, so as to establish the standard operating process for CPKP detection by VITEK MS. Our results show that VITEK MS with MEM or IPM as a substrate can specifically screen CPKP. In addition, VITEK MS with MEM or IPM as a substrate has good sensitivity and specificity for KPC-2 type (sensitivity 100%/specificity 100%), which is the mainstream of CPKP in China and other countries, as well as for the detection of IMP-4 carbapenemase from metallo-β-lactamases. In particular, VITEK MS detection with IPM as a substrate has a very short incubation time with only 20 min in the pretreatment time and can quickly observe the disappearance of the original peak value of IPM, which is worthy of promotion in the clinical microbiology laboratory.

## Data Availability Statement

The original results of this study can be found in the manuscript and supporting information. The genome sequences have been deposited in the GenBank database under the accession number Bioproject PRJNA793616. Further inquiries can be directed to the corresponding author.

## Author Contributions

HL, WW, and JW contributed to the conception and design of the study. ZH, HL, and JC organized the database. ZH and HL adjusted the instrument parameters and conducted the experiments. HL and ZH performed the statistical analysis. HL wrote the first draft of the manuscript. WW, ZH, YL, and HL wrote sections of the manuscript. All authors contributed to the manuscript revision and approved the submitted version.

## Funding

This work was supported by the Science, Technology and Innovation Commission of Shenzhen Municipality (JCYJ20160422151142150).

## Conflict of Interest

The authors declare that the research was conducted in the absence of any commercial or financial relationships that could be construed as a potential conflict of interest.

## Publisher’s Note

All claims expressed in this article are solely those of the authors and do not necessarily represent those of their affiliated organizations, or those of the publisher, the editors and the reviewers. Any product that may be evaluated in this article, or claim that may be made by its manufacturer, is not guaranteed or endorsed by the publisher.

## Supplementary Material

Supplementary Table 1 Genotype of Carbapenemase and mCIM/eCIM results of 133 CPKP strains
